# Exome analysis of carotid body tumor

**DOI:** 10.1186/s12920-018-0327-0

**Published:** 2018-02-13

**Authors:** Anastasiya V. Snezhkina, Elena N. Lukyanova, Dmitry V. Kalinin, Anatoly V. Pokrovsky, Alexey A. Dmitriev, Nadezhda V. Koroban, Elena A. Pudova, Maria S. Fedorova, Nadezhda N. Volchenko, Oleg A. Stepanov, Ekaterina A. Zhevelyuk, Sergey L. Kharitonov, Anastasiya V. Lipatova, Ivan S. Abramov, Alexander V. Golovyuk, Yegor E. Yegorov, Khava S. Vishnyakova, Alexey A. Moskalev, George S. Krasnov, Nataliya V. Melnikova, Dmitry S. Shcherbo, Marina V. Kiseleva, Andrey D. Kaprin, Boris Y. Alekseev, Andrew R. Zaretsky, Anna V. Kudryavtseva

**Affiliations:** 1 Engelhardt Institute of Molecular Biology, Russian Academy of Sciences, Moscow, Russia; 2 Vishnevsky Institute of Surgery, Ministry of Health of the Russian Federation, Moscow, Russia; 3 National Medical Research Radiological Center, Ministry of Health of the Russian Federation, Moscow, Russia; 4 Pirogov Russian National Research Medical University, Moscow, Russia

**Keywords:** Carotid body tumor, Paragangliomas, Head and neck paragangliomas, Mutations, High-throughput sequencing, Exome

## Abstract

**Background:**

Carotid body tumor (CBT) is a form of head and neck paragangliomas (HNPGLs) arising at the bifurcation of carotid arteries. Paragangliomas are commonly associated with germline and somatic mutations involving at least one of more than thirty causative genes. However, the specific functionality of a number of these genes involved in the formation of paragangliomas has not yet been fully investigated.

**Methods:**

Exome library preparation was carried out using Nextera® Rapid Capture Exome Kit (Illumina, USA). Sequencing was performed on NextSeq 500 System (Illumina).

**Results:**

Exome analysis of 52 CBTs revealed potential driver mutations (PDMs) in 21 genes: *ARNT, BAP1, BRAF, BRCA1, BRCA2, CDKN2A, CSDE1, FGFR3, IDH1, KIF1B, KMT2D, MEN1, RET, SDHA, SDHB, SDHC, SDHD, SETD2, TP53BP1, TP53BP2,* and *TP53I13*. In many samples, more than one PDM was identified. There are also 41% of samples in which we did not identify any PDM; in these cases, the formation of CBT was probably caused by the cumulative effect of several not highly pathogenic mutations. Estimation of average mutation load demonstrated 6–8 mutations per megabase (Mb). Genes with the highest mutation rate were identified.

**Conclusions:**

Exome analysis of 52 CBTs for the first time revealed the average mutation load for these tumors and also identified potential driver mutations as well as their frequencies and co-occurrence with the other PDMs.

**Electronic supplementary material:**

The online version of this article (10.1186/s12920-018-0327-0) contains supplementary material, which is available to authorized users.

## Background

Carotid body tumors (CBTs) are neoplasms of the paraganglia located at the bifurcation of carotid arteries and belong to head and neck paragangliomas (HNPGLs). These highly vascularized tumors originate in the neural crest and are typically benign. However, in 10–15% of cases the tumors can become malignant, characterized by multifocal growth, metastases, and relapse after surgical treatment. Typically, CBTs are slow-growing tumors. Surgical treatment remains high-risk and extremely challenging due to the location of the tumor in close proximity of important nerves and blood vessels. Potential damage to carotid arteries can result in cerebral circulation dysfunction [1]. One of the main difficulties in CBT management is the identification of tumors that are predisposed to be aggressive, since morphological criteria alone are not sufficiently informative [2]. To create more specific treatment strategies, there is a growing need for identification of new biomarkers that can distinguish between indolent and aggressive metastatic forms of CBTs. Many germline and somatic mutations are involved in progression of paragangliomas (PGLs). Up to 30–40% of all HNPGLs are hereditary [3, 4]. The majority of these familial paraganglioma syndromes are due to mutations in genes encoding distinctive subunits of the mitochondrial succinate dehydrogenase (SDH) complex [5]. The SDH complex participates in both the citric acid cycle and the electron transport chain, playing an essential role in energy metabolism. Besides, somatic mutations in more than 30 causative genes are described as drivers for paragangliomas [6–9].

The Cancer Genome Atlas (TCGA) consortium published the results of comprehensive molecular profiling including exome, mRNA, and miRNA sequencing, epigenomic analysis as well as reverse phase protein array for 173 pheochromocytoma (PCC) and paraganglioma tumors [10]. Four molecular subtypes were revealed in this work. Unfortunately, HNPGLs were not included in the analysis because such tumors are often embolized prior to surgery, leaving excessive necrotic tumor tissue that is insufficient for molecular analysis. The majority of researches include PGLs from many localizations and PCC in a single group, therefore it is impossible to determine the frequency of mutations in particular genes in tumors with different localizations [2, 11]. This work is dedicated to exome analysis of carotid paragangliomas for understanding the difference in causative gene set and frequency of mutations with other PGLs.

Somatic mutations occur in all cancer types [12]. In some of them, a part of somatic mutations is generated by exposures such as tobacco smoking in lung cancer and ultraviolet light in cutaneous melanoma [13], or by abnormalities of DNA maintenance as a defective DNA mismatch repair in some colorectal tumors [14]. Different mutational processes often generate different combinations of mutation types termed “signatures” [15]. Using high-throughput sequencing technology, thousands of somatic mutations can be identified in each tumor sample, and it was demonstrated that it is not correct to include only “driver” mutations in the analysis of signatures of mutational processes and mutation load (ML) [12]. Therefore, the sequencing of blood or adjusted normal tissue is used for revealing germline mutations, except ML analysis. Variant filtering by frequency in healthy population can be used to exclude potentially germline mutations in absence of non-tumor tissue. Then ML is estimated as a number of mutations per megabase (Mb) of coding regions. It was recently observed that the samples with high mutation load among the other samples in the same cancer type are more sensitive to immunotherapy, which can be very promising for tumor treatment [16–19]. This is the first reported data on mutation load and its range in CBTs.

## Methods

### Tissue sample collection and nucleic acid isolation

The paraffin-embedded tissues of 52 CBTs were collected at the Vishnevsky Institute of Surgery, Ministry of Health of the Russian Federation. The sections from paraffin-embedded tissues were prepared on glass slides and then stained with hematoxylin-eosin (H&E). Tumor areas were chosen in unstained sections according to the reference H&E slide and transferred into microcentrifuge tubes. DNA was isolated using the High Pure FFPET DNA Isolation Kit (Roche, Switzerland). DNA concentrations were measured using NanoDrop 1000 spectrophotometer (Thermo Fisher Scientific, USA) in addition to Qubit 2.0 fluorimeter (Thermo Fisher Scientific). DNA quality was estimated using the Agilent 2100 Bioanalyzer (Agilent Technologies, USA). The cohort consisted of Russian citizens with both familial and sporadic forms, 32–80 years, predominantly women (61%) requested for surgical treatment of CBTs. Patient’s consent for the study was obtained, and the protocol for investigation was approved by the ethical committee according to the preliminary discussion and agreement.

### Library preparation, sequencing, and analysis

Library preparation for exome sequencing was carried out using the Nextera® Rapid Capture Exome Kit (Illumina, USA). Sequencing was performed using NextSeq 500 System (Illumina). At least 60 million paired-end reads of 75 nucleotides in length were obtained for each sample with 300× minimal coverage.

Raw data from high-throughput sequencing in *.fatsq.gz format were aligned on hg19 reference human genome using bwa mem software. The resulting files of *.bam format were sorted and deduplicated using the SAMtools program package. Mutation calling was performed with freebayes software with filtration of identified variants with vcffilter of vcflib program package. Annotation of variants was performed using SnpSift of snpEff program package. Databases dbSNP, dbNSFP, ClinVar, MutationTaster, SIFT, PolyPhen-2, FATHMM, phastCons, PhyloP, 1000 Genomes Project, ExAC, COSMIC, GO, ConsensusPathDB, and OMIM were used as information resources for identified variants.

List of genes, which can have potential driver mutations (PDMs) involved in PGL formation, was created based on detailed literature analysis including exome sequencing results of TCGA project (http://cancergenome.nih.gov/) [2, 10]. Then PDMs in selected genes were analyzed for 52 CBT samples and characterized. We determined all non-synonymous mutations and then applied a set of filters to discriminate mutations that can be drivers for CBTs (Additional file 1). We did not discriminate somatic and germline mutations at this step because both of them can cause PGLs in a similar way, as well as we did not have access to blood or matched non-tumor tissue for many FFPE samples collected since 1990.

Mutation load analysis was performed for all samples. Variants observed in healthy population were excluded as potentially germline mutations. Therefore, we used stringent parameters for filtration, and potentially somatic deleterious mutations were counted to estimate their number per megabase of coding regions [12].

## Results

**Fig. 1:**
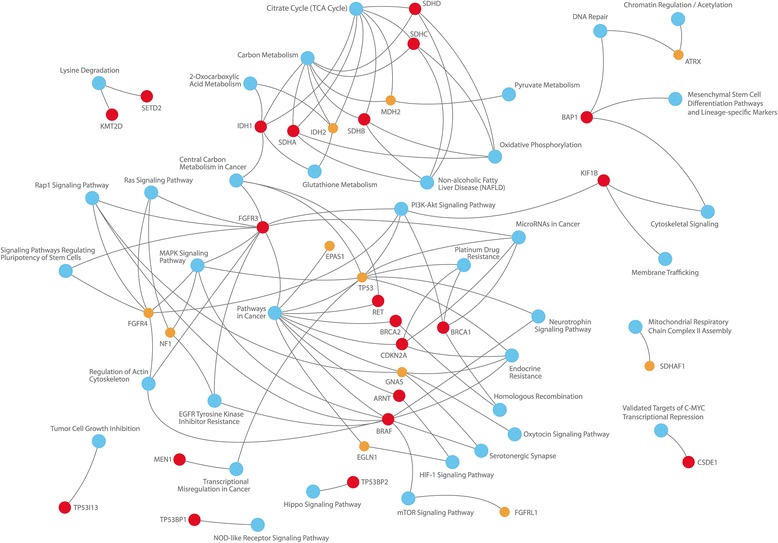
Conditions associated with the selected genes

**Fig. 2:**
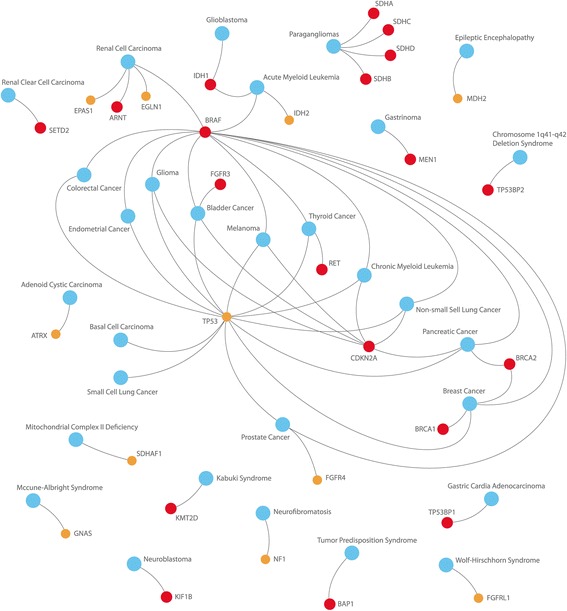
Pathways associated with the selected genes

**Fig. 3:**
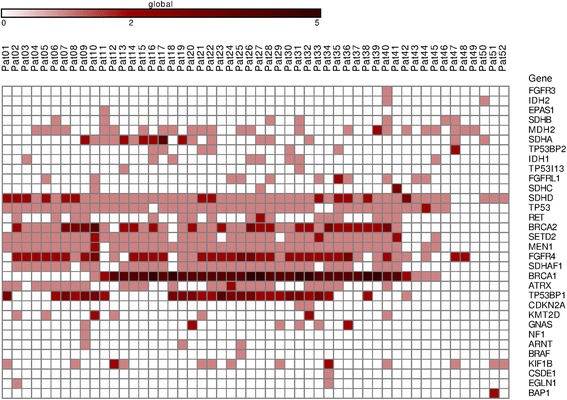
Distribution of high impact mutations in the genes of interest across the CBT samples. The list of mutation types, which belong to high impact category, is represented in Additional file 2. Colored scale indicates the number of mutations in one gene

**Fig. 4:**
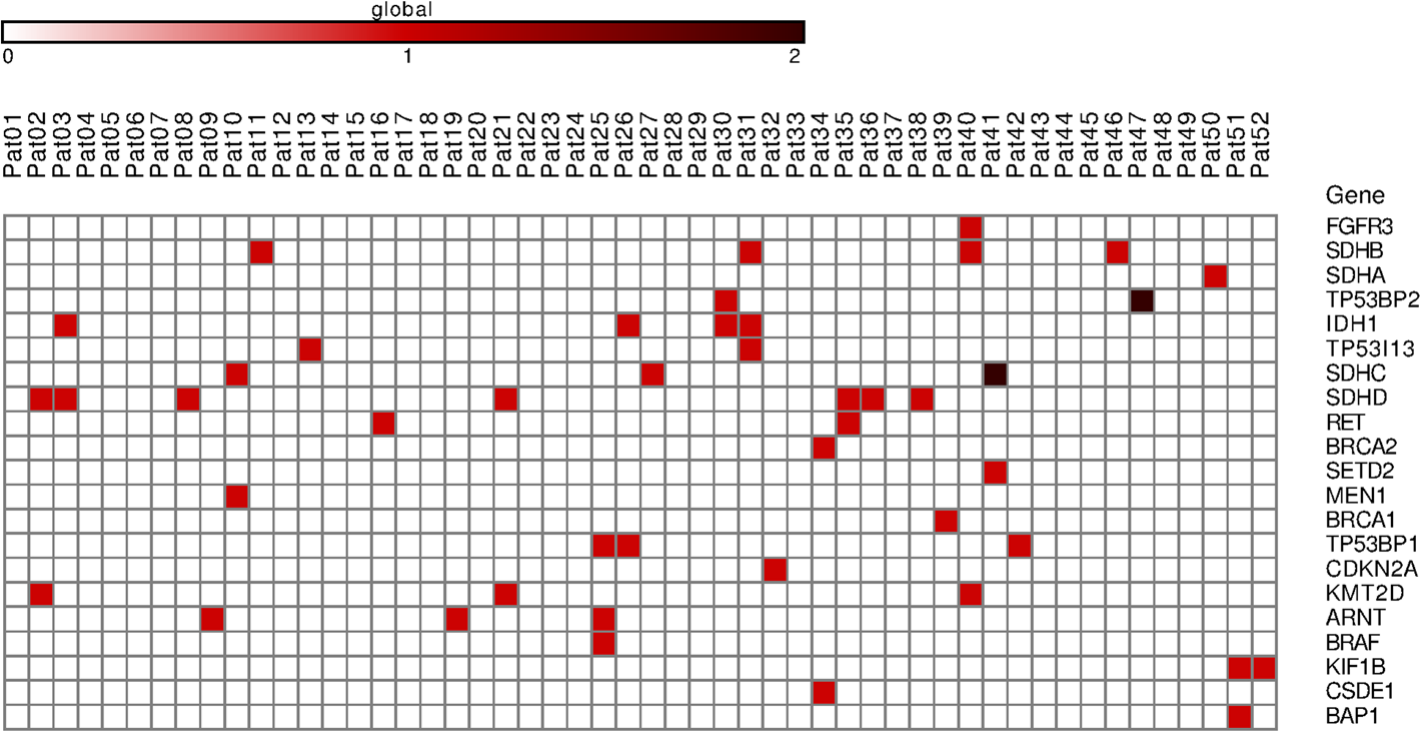
Distribution of potential driver mutations in the genes of interest across the CBT samples. For PDM determination, we apply three additional filters for the previous list. We included mutations occurring only in healthy human population with less than 2% frequency according to the ExAC database and 1000 Genomes project. Only mutations reported as benign according to ClinVar were removed. We suggested that PDMs are only those mutations which are located in conservative DNA fragments. Fragments with conservative score higher than 0.6 (scale: from 0 - highly variable fragment, to 1 - highly conservative fragment) according to phastCons for three groups (46 placentals, 46 primates, and 100 vertebrates) were included. Colored scale indicates the number of mutations in one gene

The list of 42 potentially causative genes for PGLs was created: *VHL*, *SDHA*, *SDHB*, *SDHC*, *SDHD*, *NF1*, *RET*, *HRAS*, *KRAS*, *EPAS1* (*HIF2A*), *ATRX*, *CSDE1*, *BRAF*, *FGFR1*, *FGFR2*, *FGFR3*, *FGFR4*, *FGFRL1*, *SETD2*, *ARNT*, *TP53*, *TP53BP1*, *TP53BP2*, *TP53I13*, *KMT2D*, *BAP1*, *IDH1*, *IDH2*, *SDHAF1*, *SDHAP2*, *FH*, *EGLN1*, *MDH2*, *TMEM127*, *MAX*, *KIF1B*, *MEN1*, *GDNF*, *GNAS*, *CDKN2A*, *BRCA1*, and *BRCA2*. Selected genes are involved in many important pathways including TCA cycle, DNA repair, oxidative phosphorylation, HIF-1 signaling pathway, Ras signaling pathway, PI3K-Act signaling pathway, carbon metabolism, etc. (Fig. 1, Additional file 2). Moreover, many of them are causative for different cancer types and other diseases (Fig. 2, Additional file 2). These genes were analyzed in CBTs, and high impact mutations were identified in 32 genes. Potential driver mutations were found only in 21 of them: *ARNT, BAP1, BRAF, BRCA1, BRCA2, CDKN2A, CSDE1, FGFR3, IDH1, KIF1B, KMT2D, MEN1, RET, SDHA, SDHB, SDHC, SDHD, SETD2, TP53BP1, TP53BP2,* and *TP53I13.* In 27% of CBT samples, more than one potential driver mutation was observed. Two PDMs were found in the same gene in two samples. The results are summarized in Fig. 3, Fig. 4, Table 1, and Additional file 3.

**Table 1:** Frequency of potential driver mutations (PDMs) in the selected genes, and their co-occurrence in carotid body tumor (CBT) samples

Gene	Frequency of samples with mutations, %	Associations with mutations in the other genes
*SDHD*	13.5	*RET, KMT2D, IDH1*
*IDH1*	7.7	*SDHB, SDHD, TP53BP1, TP53BP2, TP53I13*
*SDHB*	7.7	*IDH1, FGFR3, TP53I13, KMT2D*
*ARNT*	5.8	*BRAF, TP53BP1*
*SDHC*	5.8	***SDHC*** *, SETD2,MEN1*
*KMT2D*	5.8	*SDHD, SDHB, FGFR3*
*TP53BP1*	5.8	*ARNT, BRAF, IDH1*
*TP53BP2*	3.8	***TP53BP2*** *, IDH1*
*TP53I13*	3.8	*IDH1, SDHB*
*RET*	3.8	*SDHD*
*KIF1B*	3.8	*BAP1*
*CDKN2A*	1.9	*–*
*BRAF*	1.9	*ARNT, TP53BP1*
*BRCA2*	1.9	*CSDE1*
*BAP1*	1.9	*KIF1B*
*CSDE1*	1.9	*BRCA2*
*SETD2*	1.9	*SDHC*
*MEN1*	1.9	*SDHC*
*FGFR3*	1.9	*SDHB, KMT2D*
*BRCA1*	1.9	*–*
*SDHA*	1.9	*–*

Genes which have two mutations in one sample are indicated with bold

### *SDHx*

Mutations in genes encoding succinate dehydrogenase (SDH) subunits can cause pseudohypoxic state in PGLs and PCC. SDH comprises a mitochondrial protein complex that participates in both the Krebs cycle and the electron transport chain. In the Krebs cycle, this complex oxidizes succinate to fumarate, whereas, in the transport chain, it transfers electrons onto coenzyme Q [20]. The SDH enzyme complex consists of four subunits: *SDHA, SDHB, SDHC,* and *SDHD*, which form the core of the complex as well as its structural fastening elements [21]. Two more factors are also involved in the assembly of the complex: SDHAF1 [22] and SDHAF2 [23]. Therefore, a mutation in any of these genes, collectively termed *SDHx* genes, would impair the structure of the entire complex leading to oncogenesis [24]. Generally, germline mutations in the *SDHx* genes lead to heritable PGLs, PCC, and other tumors.

### *SDHA*

Only one potential driver missense mutation NM_004168: c.792C > A, p.(Phe264Leu) (chr5:231,012) was identified in *SDHA* gene. Information on this mutation in dbSNP, ClinVar, Cosmic, and Ensembl databases is absent, but predictor programs demonstrate high probability for it to be pathogenic one.

### *SDHB*

We identified four mutations in *SDHD* gene. PDM NM_003000: c.541-2A > G (chr1:17,350,571, rs786201161) is located in splice-site described in ClinVar as germline mutation with pathogenic or probably pathogenic clinical significance. It was found in different phenotypic conditions including hereditary cancer-predisposing syndrome, PGLs and PCC [25, 26], leukoencephalopathy [27], as well as kidney cancer [28].

A mutation NM_003000: c.724C > T, p.(Arg242Cys) (chr1:17,349,144, rs786203251) is deposited as germline pathogenic one and described as a risk factor for PGLs, PCC, and hereditary gastro-intestinal stromal tumor. A mutation NM_003000: c.763A > T, p.(Lys255*) (chr1:17,349,105) is included only in Ensembl database.

Two mutations, NM_003000: c.233A > G, p.(Lys78Arg) (chr1:17,359,608, rs774960237) and NM_003000: c.763A > T, p.(Lys255*) (chr1:17,349,105), do not have description of clinical effect in databases. In sample “Pat11” (Fig. 4), only the *SDHB* mutation c.763A > T was identified among selected genes of interest. It is located in the last codon of the second to last exon and forms stop-codon. However, it is indicated as non-pathogenic mutation by the SIFT predictor resource that can be explained by the algorithm of prediction ignoring changes in the end of protein.

Mutation c.233A > G has a very low frequency in population (0.000008/1, according to ExAC) and is probably germline one. According to SIFT resource, this mutation is not pathogenic. In sample “Pat31”, it co-occurs with PDMs in *IDH1* and *TP53I13* genes and probably acts as the initial driver which needs to be activated with other pathogenic influences such as additional somatic mutations.

### *SDHC*

We identified three samples with *SDHC* mutations, one of them contained two different PDMs, NM_003001: c.224G > A, p.(Gly75Asp) (chr1: 161,310,428, rs786205147) and NM_003001: c.7G > A, p.(Ala3Thr) (chr1:161,284,202, rs748243732). PDM c.224G > A is described in dbSNP as germline mutation, and it is indicated in ClinVar as probably pathogenic one, because it was found as a rare single nucleotide variant (SNV) in patient with Karney’s triad (it seems to be a sporadic disease). PDM c.7G > A was described as a rare germline mutation with unknown clinical significance in gastro-intestinal stromal tumors and PGLs. According to SIFT, its parameter is close to a threshold of 0.08 (pathogenic < 0.05). Besides, the mutation is predicted as pathogenic one by PolyPhen-2 and LRT (likelihood ratio test) and is located in a highly conservative DNA region by phastCons prediction. However, MutationTaster predicts it as non-pathogenic mutation. Therefore, we can suggest that patient has non-highly pathogenic mutations in both copies of *SDHB* or in the same copy that caused cumulative effect and became the reason for tumor growth.

A nonsense mutation NM_003001: c.183G > A, p.(Trp61*) (chr1:161,310,387) was observed in one sample. It was described only in HGMD resource (CM092024), and its clinical significance was not indicated. However, the stop-codon in the middle of ORF suggested it being pathogenic mutation.

One deletion undescribed in databases NM_003001: c.409delT, p.(Trp137fs) (chr1:161,332,121) was identified in gene *SDHC*. It leads to frameshift, which is a strong prediction factor of pathogenicity. Moreover, the stop-codon located near the frameshift in codon 133 (p.(Arg133Ter)) is represented in databases and associated with PGLs.

### *SDHD*

We identified *SDHD* mutations in seven CBT samples. In two samples, it was a nonsense mutation NM_003002: c.112C > T, p.(Arg38*) (chr11:111,958,640, rs80338843). It is described in databases as a germline pathogenic mutation associated with hereditary paragangliomas, pheochromocytoma, as well as gastric stromal sarcoma [4, 5].

A mutation NM_003002: c.205G > T p.(Glu69*) (chr11:111,959,626) was found in one of the samples. Its description in NCBI and Ensembl databases is absent, but another mutation in the same codon is deposited in Cosmic and ClinVar as germline pathogenic one [29] (NM_003002: c.205G > A, p.(Glu69Lys)). Thus, a mutation c.205G > T forming stop-codon also appears to be pathogenic one that is confirmed by data from SIFT, MutationTaster, LRT, and phastCons resources.

Mutation NM_003002: c210G > C, p.(Arg70Ser) was found in one sample. Data from NCBI and Ensembl resources are absent. A similar mutation c209G > A, p.(Arg70Lys) in the same codon is described in ClinVar with unknown clinical significance. It is deposited in Cosmic database as germline pathogenic mutation according to FATHMM data, which is also supported by all prediction resources: SIFT, PolyPhen-2, MutationTaster, and LRT. Moreover, it is located in highly conservative DNA region by phastCons information. This fact may suggest the pathogenic effect of mutation c210G > C.

Three mutations causing frameshift, that usually has a highly pathogenic effect, were evaluated – NM_003002: c.217dupA, p.(Ser73fs) (chr11:111,959,637), NM_003002: c.220_228delGTT TTG CTCinsT, p.(Val74fs) (chr11:111,959,639), and NM_003002: c.13dupT, p.(Trp5fs) (chr.11: 111,957,643). Germline mutation in the same codon c.14G > A, leading to hereditary PCC, was described earlier [4].

### *IDH1*

In the TCA cycle, IDH comprises the oxidative decarboxylation enzyme that converts isocitrate to α-ketoglutarate. Under conditions of hypoxia or in tumor cells with defective mitochondria, this enzyme participates in the reductive carboxylation of α-ketoglutarate to isocitrate during glutamine-dependent lipogenesis [30].

We found mutation NM_005896: c.548A > G, p.(Tyr183Cys) (chr2:209,108,301, rs34599179) in two samples and mutation NM_005896: c.94 T > G, p.(Phe32Val) (chr2:209,116,182, rs142923780) in one sample. Both SNVs were deposited as germline ones with uncertain clinical significance and phenotype. In one sample, we detected mutation NM_005896: c.394C > T, p.(Arg132Cys) (chr2:209,113,113, rs121913499), which is indicated in ClinVar as a somatic pathogenic/likely pathogenic variant detected in many tumor types. The found mutations change the protein structure.

The frequency of *IDH1* mutations in PCC/PGLs is low. In paragangliomas, a mutation in *IDH1* was first recorded during the analysis of 365 samples [31]. Somatic mutation in this gene was detected in carotid paraganglioma, although no *IDH* mutations were found in pheochromocytoma. According to the data of TCGA consortium, only one *IDH1* somatic mutation was identified among 173 samples in codon 132. However, it can be explained by the fact that head and neck paragangliomas were not included in the final TCGA cohort. In another study, the analysis of 104 PCC/PGLs did not reveal any mutation in *IDH1* and *IDH2* genes [32]. Therefore, we can suggest that frequency of *IDH1* mutations may be associated with localization of paragangliomas.

### *ARNT*

Gene *ARNT* encodes protein that is involved in activation of the xenobiotic metabolism genes [33]. This protein is also a cofactor for regulation of *HIF1A* transcription.

We found a rare (0.0074) variant NM_001668: c.1551 T > G, p.(Asp517Glu) (chr1:150,789,864, rs10305741) of *ARNT *gene in two samples. Data from ClinVar resources are absent. In Uniport database, it does not change any physical and chemical properties of protein. c.1551 T > G may be non-pathogenic mutation according to SIFT database.

We found mutation NM_001668: c.2335C > G, p.(Pro779Ala) (chr1:150,784,532, rs200078254) in one sample. This mutation is described in Ensembl and dbSNP databases as non-pathogenic one that is also supported by SIFT resource. The Cosmic database describes another mutation in the same codon (c.2335C > T, p. non-pathogenic (Pro779Ser)) in lung squamous cell carcinoma. According to FATHMM, it might be pathogenic mutation. However, in the case of Pro779Ser, the charge of the amino acid residue is changing in contrast to Pro779Ala.

We identified mutation NM_001668: c.1879C > T, p.(Arg627Cys) (chr1:150,788,806) in one sample. According to examined databases, it is a rare mutation with unknown clinical effect, but it might be highly pathogenic one according to SIFT resource.

### *TP53BP1*

Gene *TP53BP1* encodes tumor suppressor p53-binding protein 1, also known as p53BP1 [34, 35]. Unstable expression of *TP53BP1* is associated with genomic instability in oncocytic follicular adenoma of thyroid [36]. It plays a key role in DNA damage response, may have a role in checkpoint signaling during mitosis, and enhances TP53-mediated transcriptional activation. It was demonstrated that *TP53BP1* is downregulated in most cases of triple-negative breast cancer [37].

In one sample, we found non-pathogenic missense mutation NM_005657: c.5242C > T, p.(Arg1748Cys) (chr15:43,705,365, rs140689367) (SIFT = 0.13), highest population MAF < 0.01 described in Ensembl database. In two samples, we found NM_005657: c.880 T > C, p.(Ser294Pro) (chr15:43,769,851, rs61751060) non-pathogenic missense mutation (SIFT = 0.36) with global MAF of 0.003 described in Ensembl database.

### *TP53BP2*

Gene *TP53BP2* encodes tumor suppressor p53-binding protein 2 (p53BP2) [38–40]. p53BP2 plays an important role in regulation of apoptosis and cell growth [41]. p53BP2 binds to wild-type p53 but not to mutant one, suggesting that p53BP2 may be involved in the ability of p53 to suppress oncogenic transformation. *TP53BP2* mRNA expression is frequently downregulated in human breast cancer [42]. Four single nucleotide polymorphisms in *TP53BP2* are significantly correlated with gastric cancer susceptibility [43]. In general, patients with high *TP53BP2* expression tend to have a longer median survival time than those with low *TP53BP2* expression [44].

In one sample, we identified mutation NM_005426: c.2932C > T, p.(Arg978Cys) (chr1:223,971,861, rs199572474) described in Ensembl database. Its clinical significance is unknown by SIFT and PolyPhen-2 resources. In another sample, we found mutation NM_005426: c.179C > T, p.(Ala60Val) (chr1:223,991,959, rs61749337) described in dbSNP and Ensembl databases. The mutation is pathogenic one by MutationTaster, LRT, and PolyPhen-2 predictions (MutationTaster score = 1.0, LRT prediction = ‘Disease’, and PolyPhen-2 score = 0.94), but it has not been described in literature (Global MAF < 0.003). In one sample, we found mutation NM_005426: c.1515G > C, p.(Gln505His) (chr1:223,985,963, rs61824007) described in dbSNP and Ensembl databases. This mutation is pathogenic one by PolyPhen-2 resource (0.993), but it is tolerant by SIFT (Global MAF = 0.003).

### *TP53I13*

Gene *TP53I13* encodes tumor protein p53 inducible protein 13 that may act as a tumor suppressor, because it inhibits tumor cell growth when overexpressed. It can be upregulated by genotoxic stresses of adriamycin and/or UV irradiation in a p53/TP53-dependent manner [45].

In two samples, we found somatic missense mutation NM_138349: c.148C > G, p. (Pro50Ala) (chr17:27,896,342, rs112563021) with allele frequency 0.005810, MAF **<** 0.01(G).

### *RET*

Protein RET constitutes a transmembrane tyrosine kinase receptor for extracellular signaling molecules of the GDNF family. It is required for development of the sympathetic, parasympathetic, as well as enteric nervous systems [46]. Germline and somatic mutations occur in gene *RET* with similar frequency (approximately 5–6%) [10, 47].

We identified two samples with *RET* mutations. In both cases, it was NM_020630: c.2372A > T, p.(Tyr791Phe) (chr10:43,613,908, rs77724903) located in exon 13 and changing protein structure. This mutation is described both as germline and somatic one. Some data indicates that *RET* c.2372A > T is associated with familial medullary thyroid carcinoma (FMTC), hereditary cancer-predisposing syndrome, Hirschsprung disease, multiple endocrine neoplasia (MEN) including types 1, 2a, 2b, and 4, pheochromocytoma, and renal adysplasia [4, 48, 49].

It was observed in the work of TCGA consortium that *RET* mutations occurred in distinct protein coding regions with germline mutations clustered at codon 634 in the extracellular domain and somatic mutations clustered at codon 918 in the intracellular tyrosine kinase domain in PCC/PGLs [10]. In addition, a similar pattern was demonstrated for medullary thyroid carcinoma [50]. Mutation c.2372A > T is also located in tyrosine kinase domain, but it is deposited in ClinVar database several times as both germline and somatic one.

### *KIF1B*

The *KIF1B* gene consists of 50 exons and encodes two protein isoforms, KIF1Bα and KIF1Bβ. These proteins are involved in anterograde transport of mitochondria (KIF1Bα) and synaptic vesicle precursors (KIF1Bβ) [51, 52]. In addition, KIF1Bβ was shown to represent a target of PHD3 protein and is involved in apoptosis. Missense mutations in the *KIF1B* gene were found for the first time in two PCC samples in 2008 [53]. Later, a group of relatives was found that increased probability of developing not only PCC, but also neuroblastomas, ganglioneuromas, and lung tumors for germline mutations in the *KIF1B* gene [54]. No mutations in the KIF1B gene have been detected in PGLs. In this work, we identified two *KIF1B* mutations NM_015074: c.4717C > T, p.(Pro1573Ser) (chr1:10,431,229) and NM_015074: c.1579G > C, p.(Gly527Arg) (chr1:10,355,764).

Both germline and somatic mutations in *KIF1B* gene have been found in pheochromocytoma, occasionally occurring in combinations with mutations in other genes, such as *NF1*, *RET, VHL,* and *SDHx* [54, 55]. In our PGL cohort, one of two samples also has additional PDM in *BAP1* gene.

### *CDKN2A*

The *CDKN2A* gene is located on the long arm of chromosome 9 at position 21.3, and encodes several proteins. The most well-studied proteins are p16 (INK4a) and p14 (ARF). Both proteins act as tumor suppressors. p16 protein in complex with CDK4 and CDK6 provides cell cycle regulation. p14 protein protects p53 and, therefore, can prevent malignization. *CDKN2A* gene is frequently mutated in head and neck squamous cell carcinomas.

In one sample, we identified mutation NM_058197: c.187G > C, p.(Gly63Arg) (chr9:21,974,640, rs45456595). According to ClinVar, this germline mutation has a conflicting clinical significance of pathogenicity. The occurrence frequency of c.187G > C mutation is 0.001.

### *BRAF*

Protein BRAF belongs to the family of RAF serine/threonine kinases and participates in activation of the RAS/RAF/ERK signaling pathway [56]. Mutations in *BRAF* gene were initially identified in cancer types that are commonly associated with mutations in different isoforms of RAS, such as malignant melanoma and well-differentiated thyroid cancer.

We identified only one sample with a non-canonical *BRAF* mutation NM_00433: c.533G > A, p.(Arg178Gln) (chr7:140,508,767, rs746348396), which changes protein structure. It has been deposited in ClinVar only once as the germline mutation with uncertain clinical significance in patient with “rasopathy” phenotype. In TCGA dataset of PGLs and PCC, only one somatic *BRAF* p.(G469A) SNV and also one fusion involving *BRAF* was identified [10]. A common activating mutation in *BRAF* p.(V600E) has been recently found in one of 60 analyzed samples of PCC [57].

### *BRCA1* and *BRCA2*

These genes play an important role in DNA repair, cell cycle checkpoint regulation, and genome stability maintenance [58]. Germline mutations in these genes are associated with hereditary breast and ovarian cancers [59], Fallopian tube, prostate, peritoneal, and pancreatic cancers [60]. Germline mutations in *BRCA* genes were demonstrated for two patients with PCC [61].

PDM NM_003000: c.5019G > A, p.(Met1673Ile) (chr17:41,222,975, rs1799967) was identified in *BRCA1* gene. According to ClinVar, this mutation is non-pathogenic one. Frequency of occurrence of this mutation based on ExAC dataset is 0.0176.

In one sample, a rare mutation NM_000059: c.4585G > A, p.(Gly1529Arg) (chr13:32,913,077, rs28897728) was evaluated in *BRCA2* gene. Despite the low frequency in population (0.0004, according to ExAC), it is considered to be non-pathogenic mutation based on different investigations.

### *CSDE1*

Gene *CSDE1* (cold shock domain containing E1) encodes a RNA-binding protein. It is required for internal initiation of translation of human rhinovirus RNA. It may be also involved in translationally coupled mRNA turnover. In this gene, we identified a mutation NM_007158: c.416C > T, p.(Ala139Val) (chr1:115,277,136, rs151170620). This mutation with unknown clinical significance was not described previously. It is predicted to be pathogenic mutation by resources such as PolyPhen-2, MutationTaster, and LRT. Besides, it is a rare mutation and its frequency of occurrence in population of healthy people is 0.0001.

### *BAP1*

BAP1 represents an onco-suppressor that participates in the regulation of the cell cycle, differentiation, gluconeogenesis, and DNA damage response [62]. Germline mutations in the *BAP1* are associated with increased risks of malignant mesothelioma as well as uveal and cutaneous melanoma, whereas somatic mutations in this gene have been detected in various types of tumors including PGLs [63, 64].

We identified *BAP1* mutation NM_004656: c.1135G > A, p.(Ala379Thr) (chr3:52,438,584) in a sample. It is not represented in dbSNP, ClinVar, and Ensembl databases, but it may be pathogenic mutation based on SIFT, PolyPhen-2, LRT, and MutationTaster algorithms. Moreover, it is located in a highly conservative DNA region (phastCons resource data), and it does not occur in population of healthy people (ExAc and 1000 Genomes project data).

### *MEN1*

The *MEN1* gene encodes the menin protein, which is localized in the nucleus and interacts with different proteins involved in transcription regulation, genome stabilization, cell division, and proliferation [65, 66]. Germline mutations in the *MEN1* gene may lead to multiple endocrine neoplasia type 1 (MEN1 syndrome), and be associated with the development of over 20 types of endocrine and non-endocrine tumors including PCC and PGLs [67–70].

Mutation NM_130799: c.512G > A, p.(Arg171Gln) (chr11:64,575,505, rs607969) was determined in one sample. In ClinVar database, it is deposited as germline mutation with unknown pathogenic significance [71]. In Cosmic database, this mutation is described as germinal one in the heterozygous state in adrenal cortical carcinoma [72] and as somatic one in hemangioblastoma [73] and prostate carcinoma. According to SIFT, MutationTaster, and FATHMM, it is pathogenic mutation. Although mutations in *MEN1* gene are rare, they represent important objects of screening as they provide the means of early diagnostics of MEN1 syndrome [74].

### *SETD2*

The SETD2 protein is a histone methyltransferase that is specific for lysine-36 histone H3, and methylation of this residue is associated with chromatin activation. This protein also contains a transcriptional activation domain associated with hyperphosphorylated RNA polymerase II.

We found PDM NM_014159: c.3229A > G, p.(Thr1077Ala) (chr3:47,162,897, rs114719990) in one CBT sample. It was described as germline mutation in the article dedicated to genes of predisposition to malignancy, data is available in ClinVar. Unfortunately, there are no data about the clinical effect of this mutation [75]. Based on SIFT, MutationTaster, LRT, and PolyPhen-2 algorithms, c.3229A > G might be highly pathogenic mutation.

### *FGFR3*

This gene encodes a member of the fibroblast growth factor receptor (FGFR) family, and its amino acid sequence is highly conserved. The extracellular part of the protein interacts with fibroblast growth factors driving a cascade of downstream signals, ultimately affecting mitogenesis and differentiation. FGFR3 binds the acidic and basic fibroblast growth hormone and plays a role in the development and maintenance of bone tissue. Mutations in this gene lead to craniosynostosis and multiple types of skeletal dysplasia. Three variants of transcripts, that encode different isoforms of the protein, are described.

We evaluated PDM NM_000142:c.1150 T > C, p.(Phe384Leu) (chr4:1,806,131, rs17881656) in one sample. Mutation 1156 T > C was described as germline one with unknown clinical significance [75]. Based on data of several researchers (ClinVar) as well as on SIFT, the prediction is that this mutation is probably non-pathogenic one.

### Mutation load

**Fig. 5:**
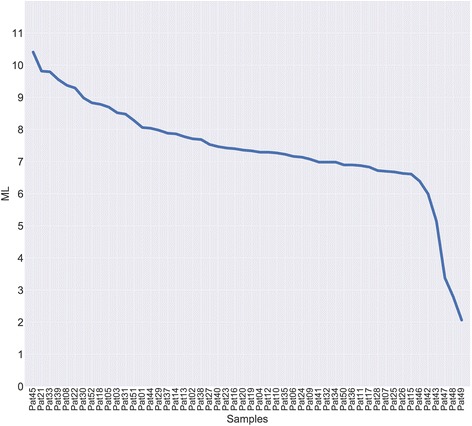
Analysis of mutation load in carotid body tumor (CBT) samples. Distribution of potentially somatic deleterious mutations among the CBT samples was evaluated to estimate their number per megabase of coding regions

**Fig. 6:**
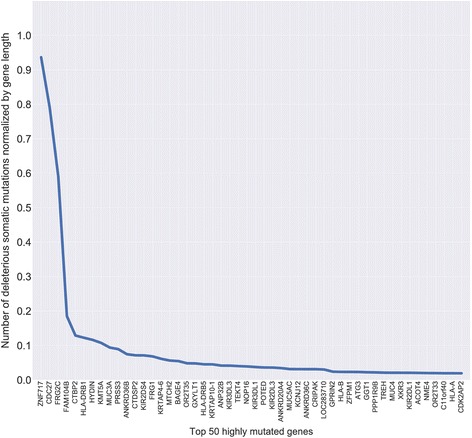
List of top 50 most frequently mutated genes in CBTs. Number of potentially somatic deleterious mutations normalized by gene length for 52 samples in total is indicated by Y-axis

**Fig. 7:**
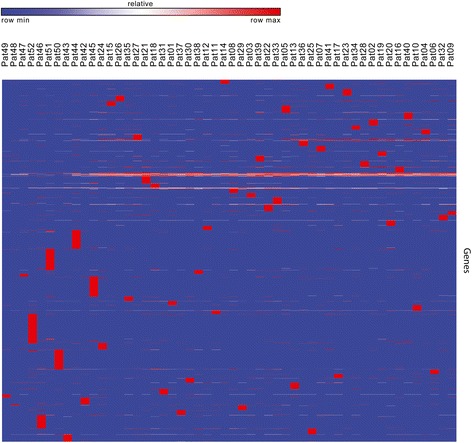
Heatmap of potentially somatic deleterious mutations in CBTs normalized by gene length across all genes for all patients. Red color indicates highly mutated genes and blue color flags genes with low level of potentially somatic deleterious mutations

Mutation load was estimated for all samples (Fig. 5, Additional file 4). The average ML was 6–8 potentially somatic deleterious mutations per megabase and varied from 2 to 10.5 mutations. Moreover, the most mutated genes were identified (Fig. 6, Additional file 5). Top 50 genes with the highest somatic mutation number are represented in Fig. 6, but only 20 genes have a very high amount of mutations and then the curve plateaus. This list includes *ZNF717, CDC27, FRG2C, FAM104B, CTBP2, HLA-DRB1, HYDIN, KMT5A, MUC3A, PRSS3, ANKRD36B, CTDSP2, KIR2DS4, FRG1, KRTAP4–6, MTCH2, BAGE4, OR2T35, GXYLT1*, and *HLA-DRB5*. Figure 7 illustrates that these genes, being highly mutated almost in all samples, are clustered and visualized in the lower part of the heatmap. The set of other highly mutated genes is different from sample to sample and seems to be specific for each tumor.

## Discussion

Carotid body tumor is the most common form of HNPGLs (60–80%), but it is not well investigated with different molecular methods, such as mRNA and miRNA analysis, as well as methylation profiling. The first reason is absence of normal matched tissue. The second one is the requirement for embolization of tumors before surgical treatment [76]. Necrotic tissue forms after this procedure, and mutational tests became the only possible molecular analysis. Thus, TCGA consortium excluded CBTs from PGLs/PCC analysis due to inability for comprehensive approach to this type of tumor, and the reliable mutation frequency for all patients with CBT is unknown [10]. Commonly, only the cohorts with familial history or the cohorts with multiple tumors, as well as with metastatic cases are included in mutational analysis [77]. It provides incorrect data on frequency of mutations in different genes across all CBT cases. For example, regarding molecular genetics, head and neck paragangliomas have been associated with nine susceptibility genes *NF1, RET, VHL, SDHA, SDHB, SDHC, SDHD, SDHAF2 (SDH5)*, and *TMEM127* [77]. Inherited HNPGLs are mostly caused by mutations in *SDHD*, *SDHB,* and *SDHC* genes. HNPGLs are rarely associated with mutations of *VHL, RET*, and *NF1*. In addition, it was shown that multiple HNPGLs are common in patients with *SDHD* mutations, while malignant head and neck paragangliomas are mostly seen in patients with *SDHB* mutations [77]. Thus, it was demonstrated that the penetrance for *SDHD* germline mutations is about twice as high as that of *SDHB* carriers. *SDHD* mutations induced HNPGLs about 20 years earlier compared with *SDHB* mutations [78]. And only correlation of germline *SDHB* mutations with tumor aggressiveness is indicated as an established fact.

For the first time exome analysis for representative cohort of 52 carotid paragangliomas, collected from the single origin (carotid body) and consisting of patients with familial and sporadic forms, was performed hereby. The frequencies of PDMs in different cancer-associated genes and mutation load were determined in CBTs from Russian patients. According to our results, the frequency of mutations in the main causative genes is different from the other investigations [10, 79]. The most common gene with PDMs was *SDHD,* 13.5% of the entire cohort (Table 1). In total, *SDHx* mutations span 26% of patients, but in some cases, *SDHx* mutations co-occur with PDMs in other genes. Mutations in *SDHx* genes increase the stability of HIFs and, thus, the expression of their targets through the intracellular accumulation of their substrate, succinate. It could determine pseudohypoxic phenotype potentially resulting in energy metabolism changes and correlate with aggressive phenotype, and it requires different management [47, 80, 81].

We did not identify mutations in *NF1, VHL, MEN1, SDHAF2,* and *TMEM127* genes, which are indicated in many papers as causative for carotid PGLs. Differences in frequency could be elucidated either by population specificity or inclusion of PGLs from different origins as well as even PCC in this cohort and their simultaneous analysis. One more reason might be the difference in filtration methods for evaluation of potentially pathogenic mutations. Future investigations will allow standardizing this procedure after depositing new mutations in databases and experimental estimation of their pathogenic effect.

In 27% of CBT samples, more than one potential driver mutation was observed. In two samples, two PDMs were observed in the same gene (*SDHC* and *TP53BP2*). It is possible that one mutation is not always enough for oncogenic cell transformation. In some cases, it could probably be explained by tumor heterogeneity. We did not identify PDMs in 44% of tumors using sufficiently strong settings for filtration. We suggest that two or more non-highly pathogenic mutations demonstrate cumulative effect and can cause tumor growth in many cases. It is critically important to be concentrated on the PDMs in other genes, which were not described before as PGLs/PCC causative.

The prevalence of somatic mutations was demonstrated to be highly variable between and within cancers, ranging from about 0.001/Mb to more than 400/Mb. The lowest ML was observed in pilocytic astrocytoma and the highest one - for malignant melanoma, lung, and bladder cancer [12]. In CBTs, we revealed ML of approximately 6-8 mutations per megabase. Although, we cannot compare our data directly with the result published in [15] because of different methods for somatic mutation identification. In mentioned paper, normal DNA from the same individuals was sequenced to establish the somatic origin of variants [12]. We used samples collected since year 1990, and in most cases the non-tumor tissues were unavailable. Thus, the computational methods for potentially germline mutation exclusion were used. Algorithms for filtration based on frequency of mutations in healthy individuals (1000 Genomes project) have been shown to exclude more than 96% of germline mutations [82]. We used additional database and applied more stringent requirements for filtration. The only variants established as somatic mutations were the ones non-existent in populations of healthy individuals according to the ExAC database and 1000 Genomes data. We considered only deleterious variants (i.e., frameshift or non-synonymous coding variants) for further analysis of ML. In order to estimate ML across all patients, we have normalized the number of potentially somatic deleterious mutations per megabase of coding region. The coding region length was 45,540,944 base pairs. To identify the most mutated genes, we divided the number of potentially somatic deleterious mutations by the length of gene coding region (gene length).

Therefore, using this type of analysis allows comparing samples within cohort to distinguish hypermutated samples. Recently, a close relationship between ML and response to checkpoint immunotherapy was demonstrated for several cancer types [83–85]. In highly mutated samples, a number of neoantigens become visible to the immune system, creating the basis for effective T cell responses. Checkpoint inhibitors, such as ipilimumab, nivolumab, and pembrolizumab, have provided a breakthrough in cancer immunotherapy including treatment of melanoma, colorectal, and lung cancers [86]. Immunotherapy will probably be a promising approach for treatment of carotid paragangliomas with high ML, especially for aggressive and metastatic tumors.

## Conclusions

Exome analysis of CBTs revealed the average mutation load for these tumors, identified potentially driver mutations as well as their frequencies and co-occurrence with other PDMs. Several novel undescribed mutations were identified. Evaluation of driver mutations leading to tumor growth, progression, and differences in clinicopathologic characteristics, as well as identification of highly mutated samples provide opportunities to develop new approaches for targeted therapy and immunotherapy of the disease, and increase the management level of CBTs.

## Additional files

Additional file 1: High impact mutation types included in the analysis. (XLSX 9 kb)

Additional file 2: Pathways and conditions associated with the selected genes. (XLSX 57 kb)

Additional file 3: List of evaluated potentially driver mutations and their detailed description. (XLSX 22 kb)

Additional file 4: Mutation load (ML) – number of computationally filtered deleterious somatic mutations per megabase of coding region across all samples. (XLSX 48 kb)

Additional file 5: Total number of deleterious somatic mutations normalized by gene length in 52 CBT samples. (XLSX 132 kb)

## Abbreviations

CBT: Carotid body tumor
HNPGLs: Head and neck paragangliomas
ML: Mutation load
PCC: Pheochromocytoma
PDM: Potential driver mutation
PGLs: Paragangliomas
*SDHx*: Genes encoding components of succinate-dehydrogenase complex. In this work: *SDHA, SDHB, SDHC, SDHD, SDHAF1*, and *SDHAF2*
